# Effects of High-intensity Robot-assisted Hand Training on Upper Limb Recovery and Muscle Activity in Individuals With Multiple Sclerosis: A Randomized, Controlled, Single-Blinded Trial

**DOI:** 10.3389/fneur.2018.00905

**Published:** 2018-10-24

**Authors:** Marialuisa Gandolfi, Nicola Valè, Eleonora Kirilova Dimitrova, Stefano Mazzoleni, Elena Battini, Maria Donata Benedetti, Alberto Gajofatto, Francesco Ferraro, Matteo Castelli, Maruo Camin, Mirko Filippetti, Carola De Paoli, Elena Chemello, Alessandro Picelli, Jessica Corradi, Andreas Waldner, Leopold Saltuari, Nicola Smania

**Affiliations:** ^1^Department of Neurosciences, Biomedicine and Movement Sciences, University of Verona, Verona, Italy; ^2^The BioRobotics Institute, Scuola Superiore Sant' Anna, Polo Sant' Anna Valdera, Pontedera, Italy; ^3^Section of Neuromotor Rehabilitation, Department of Neuroscience, ASST Carlo Poma, Mantova, Italy; ^4^Centro di riabilitazione Franca Martini—ATSM ONLUS, Trento, Italy; ^5^UOC Neurorehabilitation, AOUI Verona, Verona, Italy; ^6^Department of Neurological Rehabilitation, Private Hospital Villa Melitta, Bolzano, Italy; ^7^Research Department for Neurorehabilitation South Tyrol, Bolzano, Italy; ^8^Department of Neurology, Hochzirl Hospital, Zirl, Austria

**Keywords:** upper limb abnormalities, quality of life, rehabilitation, robotics, electromyography, learning

## Abstract

**Background :** Integration of robotics and upper limb rehabilitation in people with multiple sclerosis (PwMS) has rarely been investigated.

**Objective:** To compare the effects of robot-assisted hand training against non-robotic hand training on upper limb activity in PwMS. To compare the training effects on hand dexterity, muscle activity, and upper limb dysfunction as measured with the International Classification of Functioning.

**Methods:** This single-blind, randomized, controlled trial involved 44 PwMS (Expanded Disability Status Scale:1.5–8) and hand dexterity deficits. The experimental group (*n* = 23) received robot-assisted hand training; the control group (*n* = 21) received non-robotic hand training. Training protocols lasted for 5 weeks (50 min/session, 2 sessions/week). Before (T0), after (T1), and at 1 month follow-up (T2), a blinded rater evaluated patients using a comprehensive test battery. Primary outcome: Action Research Arm Test. Secondary outcomes: Nine Holes Peg Test; Fugl-Meyer Assessment Scale–upper extremity section; Motricity Index; Motor Activity Log; Multiple Sclerosis (MS) Quality of Life−54; Life Habits assessment—general short form and surface electromyography.

**Results:** There were no significant between-group differences in primary and secondary outcomes. Electromyography showed relevant changes providing evidence increased activity in the extensor carpi at T1 and T2.

**Conclusion:** The training effects on upper limb activity and function were comparable between the two groups. However, robot-assisted training demonstrated remarkable effects on upper limb use and muscle activity. https://clinicaltrials.gov NCT03561155.

## Introduction

Multiple sclerosis (MS) is the most common non-traumatic cause of neurologic disability in young adults worldwide (1). The major causes of disability are inflammatory demyelination and axonal loss^2^, which result in the hallmark motor, sensory, cognitive, and autonomic dysfunctions found in people with MS (2, 3). In the first year of disease onset up to 66% of patients will develop upper limb impairment that will continue to worsen over the following three decades (3–5) and diminish participation and quality of life (5, 6) Temporal fluctuations and fatigue render clinical management extraordinarily complex (3).

In the last decade, integration of robot-assisted devices in upper limb training programs has gained increasing interest for their capability to provide early, intensive, task-specific and multisensory stimulation especially in stroke patients (7). There is consensus on the effectiveness of upper limb rehabilitation also in people with MS (8). In their review, Lamers et al. emphasized the importance of multidisciplinary rehabilitation to improve upper limb capacity, along with body function and they suggested that upper limb capacity could be enhanced by robot-assisted training (8). Despite differences in sample characteristics and methodologies, the literature generally supports the benefits of upper limb robot-assisted training in people with MS. However, studies differ considerably in primary outcomes (activity vs. function), study design [uncontrolled vs. randomized controlled trial [RCT]], and therapy content and dosage. Only two controlled trials on upper limb robot-assisted training in people with MS have used devices designed for rehabilitating the proximal upper limb (shoulder and elbow) (9, 10). No studies to date have been performed using a robot-assisted device specifically designed for the hand in people with MS.

The Amadeo®(Tyromotion-Austria) is a modern, mechatronic end-effector robotic device. Its most distinctive feature is that it simulates natural grasping motion and executes automated movement sequences. Results from its application in stroke rehabilitation suggest that robot-assisted hand rehabilitation reduces motor impairment and increases use of the affected hand, with possible generalization to the entire upper limb (11, 12). It is not clear, however, whether these improvements can translate to increased upper limb use in everyday activities (11).

The primary aim of this study was to compare the effects of robot-assisted hand training and robot-unassisted rehabilitation on upper limb activity. The secondary aim was to compare the training effects on hand dexterity and upper limb function, disability, and quality of life. We hypothesized that, because it boosts greater use of the hand, upper limb activity would improve more after robot-assisted hand training than after non-robotic training. To explore the potential mechanisms involved in such improvements the electromyographic activity of 6 upper limb muscles was investigated. Given the multiplicity of symptoms that often need to be addressed in MS, the integration of robotics and rehabilitation holds promise for developing high-intensity, repetitive, task-specific, interactive treatment of upper limb impairment.

## Materials and methods

### Trial design

This single-blind RCT compared the effects of robot-assisted [experimental group (EG)] vs. non-robotic [control group (CG)] training. The examiner was blinded to group assignment (Figure 3).

### Participants

From March 2014 to March 2017, consecutive outpatients with MS and hand dexterity deficits referred to our Neurorehabilitation Unit (AOUI Verona) were assessed. Inclusion criteria were: confirmed MS diagnosis (13), age between 18 and 65 years, Expanded Disability Status Scale (EDSS) score 1.5 ≤ x ≤ 8 (13), Mini-Mental State Evaluation (MMSE) score ≥24/30 (14), Modified Ashworth Scale (MAS) score <2 evaluated at the elbow, wrist, and fingers (15), Nine Hole Peg Test (NHPT) score between 30 and 300 s (9). Exclusion criteria were: relapse or relapse-related treatments in the 3 months before entering the study, musculoskeletal impairments or visual analog scale (VAS) for pain score > 7/10 in any joint that could interfere with the training program, severe visual dysfunction, any type of rehabilitation in the month prior to recruitment, other concomitant neurological or orthopedic diseases involving the upper limb and interfering with their function. Patients gave their written, informed consent after being informed about the experimental nature of the study. The study was carried out in accordance with the Helsinki Declaration, approved by the local Ethics Committee (prog n.230CESC), and registered at clinical trial (NCT03561155).

### Interventions

One experienced physical therapist per treatment group supervised the training sessions. Patients received individualized treatment for 50 min/day, 2 days/week for 5 weeks at the physical therapy facility of the Neurorehabilitation Unit (AOUI Verona). At the end of each session, upper limb passive mobilization was performed in supine position for 10 min. Based on baseline assessment, the weaker upper limb was selected for evaluation and treatment. When both upper limbs were equally impaired, the participant's preference was taken into account.

### Experimental group

**Figure 1:**
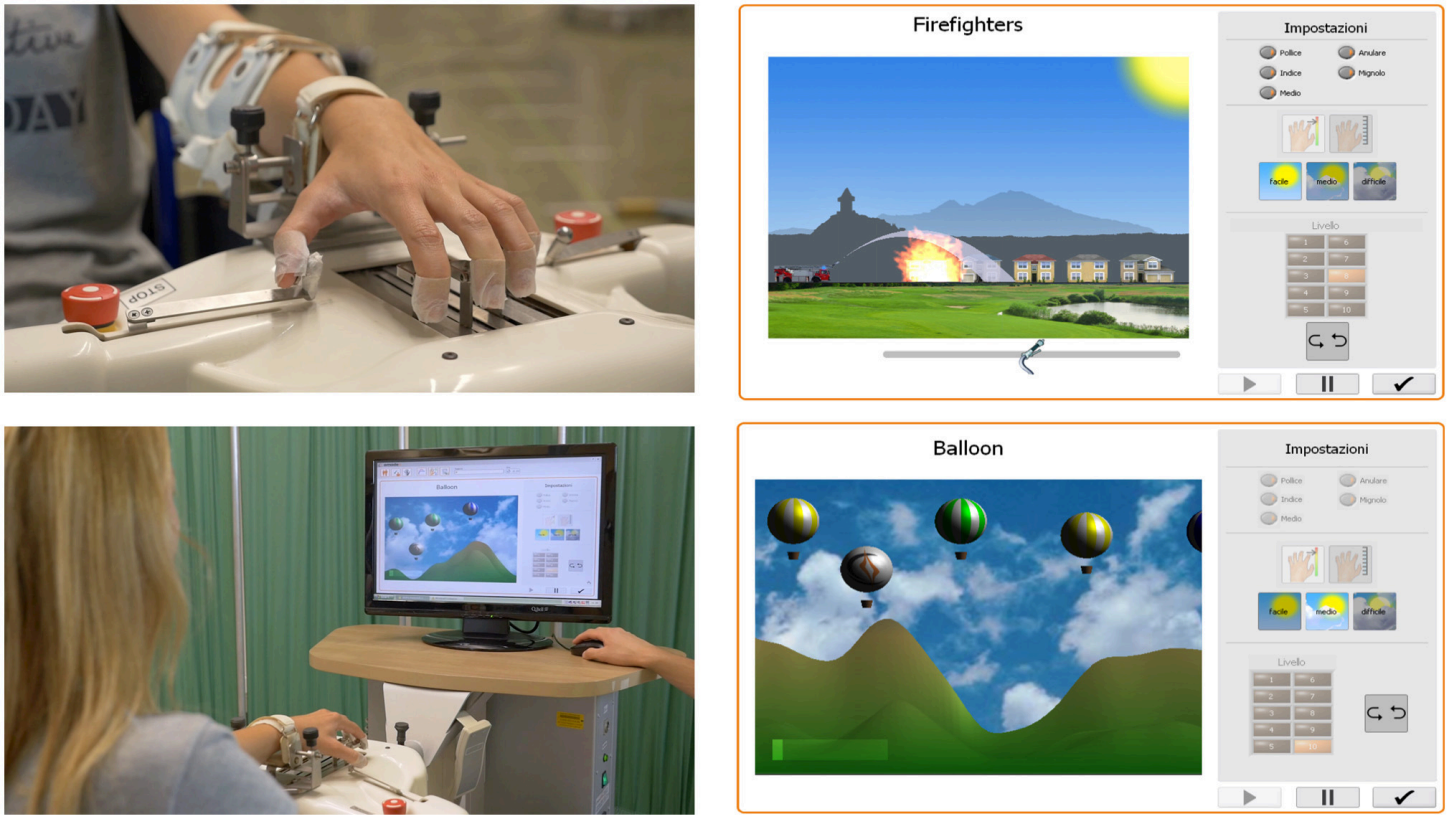
Hardware set-up of the Amadeo System, finger slings, and visual display.

Patients underwent robot-assisted hand training on an Amadeo® (Tyromotion, Austria). This modern, mechatronic end-effector computer-assisted robotic device is specifically designed to improve sensorimotor functions in patients with restricted hand function (Figure 1). Therapy sessions were conducted by a physical therapist experienced in use of the device. The patient was seated in a comfortable position and the arm strapped into an adjustable stabilizing splint attached to the robotic device, with the wrist in neutral position and the forearm pronated. The wrist was stabilized to the body of the device by means of a spring-loaded hinge, which allowed for some degree of passive flexion and extension during use. The height of the device was adjusted to an angle of about 30° of elbow flexion. Each finger was attached to the robotically driven slide with magnets taped to the distal phalanx of each finger. Three different training modes were performed: (1) continuous passive motion (CPM) during which the hand is passively stimulated in finger flexion and extension (10 min); (2) assistive therapy in which the hand is functional but is actively trained at the patient's limit of performance (10 min); (3) interactive therapy via active training with specifically developed virtual therapy games (10 min) in which the patient exerts isometric force in flexion or extension to avoid obstacles or to reach a target (fire) shown on the video. The isometric force produces proportional movement of a virtual figure. The physiotherapist sets task difficulty from among 30 pre-selected levels graded by duration, force, and accuracy in completing the task. Each exercise was repeated several times according to the patient's ability and task complexity was increased as performance improved. The physiotherapist recorded on the patient's chart the exercises (i.e., type of exercise, number of repetitions) and any adverse events that occurred during the study.

### Control group

The protocol for upper limb rehabilitation was designed according to the neurodevelopmental technique and consisted of upper limb mobilization (shoulder girdle, elbow, wrist, and finger joints), facilitation of movements, and active tasks chosen out of 15 that are challenging for patients (16, 17). The exercises were focused on improving muscle strength in flexion and extension, dexterity, and motor control. At the end of each treatment session, the patient received feedback about her/his performance in terms of the number of errors and comments on execution of movement.

### Outcomes

Demographic and clinical data (EDSS score, disease duration, and Tremor Severity Scale score) were collected at baseline (18). A comprehensive test battery was administered before (T0), after (T1), and again at 1 month of follow-up (T2).

The primary outcome was the change in upper limb activity as measured with the Action Research Arm Test (ARAT) at T1 compared to T0 (19). Secondary outcomes: upper limb activity measured using the Nine Holes Peg Test (NHPT) (20–22); manual dexterity speed calculated as the number of pegs placed per second. Trials in which patients were unable to place any peg within the time limit of 300 s were scored as 0 peg per second (6). Upper limb function was measured by means of the Fugl-Meyer Assessment Scale–upper extremity section (FMA) (range, 0–66 where higher scores indicate better performance) (23) and the Motricity Index (MI) (range, 0–100 where higher scores indicate better performance) (24). The Motor Activity Log (MAL) was used to assess changes in the amount and quality of arm use in accomplishing 30 daily activities (range, 0–168 where higher scores indicate better performance) (25). The MS Quality of Life−54 (MSQoL-54), with the physical health (PHC) and mental health (MHC) domains, were used to investigate generic and MS-specific domains of health-related QoL (range, 0–100 where higher scores indicate better performance) (26). Patient satisfaction with daily activities or social roles was assessed using the Life Habits assessment—general short form (LifeH) (27).

### Electromyography (EMG)

**Figure 2:**
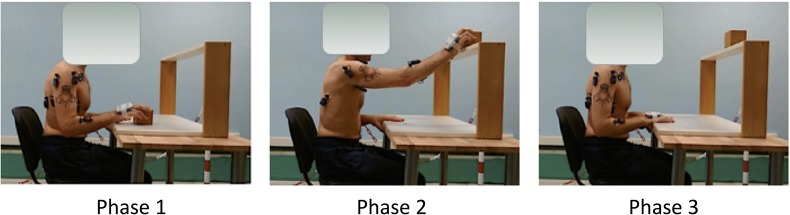
Experimental set-up with the surface EMG electrodes placement.

The EMG activity of 6 upper limb muscles of the more affected side (deltoid scapular, deltoid clavicular, triceps brachii, biceps brachii, flexor carpi radialis, and extensor carpi radialis) was measured using pairs of self-adhesive surface electrodes. Disposable Ag-AgCl electrodes were placed according to SENIAM guidelines with an inter-electrode spacing of 0.02 m. Before electrode placement, the skin was shaved with a disposable, single-use razor and cleaned with alcohol (28). Raw EMG signals were collected using BTS FREEEMG 300 wireless surface EMG sensors (BTS spa, Milan, Italy) at a sampling rate of 1000 Hz. Raw EMG signals were processed with a customized routine developed in MATLAB environment (MathWorks, USA). The raw EMG signal was bandpass filtered at 20–450 Hz and then smoothed using a 20 ms root mean square (RMS) algorithm to obtain the envelope. Signals were recorded in three conditions: 30 s during resting position (basal), 5 s of maximal voluntary isometric contraction (MVIC), and during a reach-to-grasp task (ARAT subscale). Patients were asked to grasp and place a 7.5 cm wooden-cube on a shelf of standardized height. The movement was divided in three phases by identifying 4 temporal events (start, grasping the cube, placing the cube on the shelf, returning to base position). The task was repeated 3 times and the signals were time-normalized. Normative data were collected from 14 healthy age-matched controls undergoing one surface EMG acquisition. The EMG tasks are illustrated in Figure 2.

**Figure 3:**
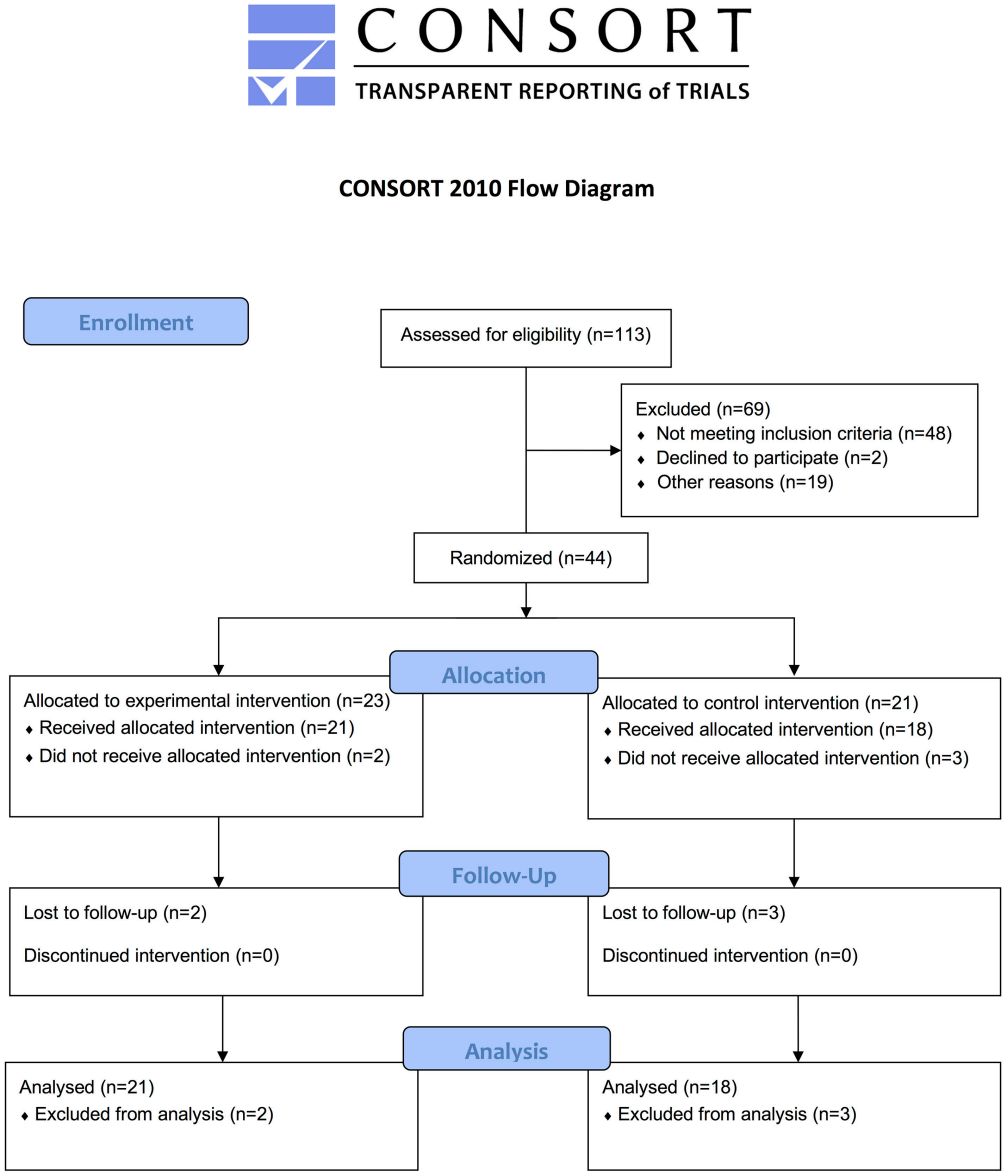
CONSORT flowchart.

### Sample size

A sample size of 36 patients (18 per group) was estimated to have 80% power to detect a mean difference of 3 on the primary outcome measure (ARAT) and an alpha (probability of type 1 error) of 5% (10). Assuming a 10% drop-out rate, 40 patients were necessary to perform the study.

### Randomization

Eligible patients were assigned to either the EG or the CG by a simple randomization scheme using an automated randomization system (www.randomization.com). Group allocation was kept concealed. The randomization list was locked in a desk drawer accessible only to the principal investigator.

### Blinding

Primary and secondary outcomes were measured by the same blinded examiner at each session.

### Statistical analysis

A per-protocol analysis was used. Descriptive statistics included means and standard deviation. The *X*^2^ test was utilized for categorical variables. Since the data were normally distributed (Shapiro-Wilk Test), parametric tests were used for inferential statistics. Two-way mixed ANOVA was applied using “Time” as the within-group factor and “Group” as the between-group factor. Two-tailed Student's *t*-test for unpaired data was used for between-group comparisons. The level of significance was set at *p* < 0.05. Bonferroni's correction was applied for multiple comparisons (*p* < 0.025). Statistical analysis was performed with SPSS 20.0 (IBM SPSS Statistics for Windows, Version 20.0, Armonk, NY, USA).

## Results

In all, 113 patients were consecutively assessed: 59 were excluded because they did not meet inclusion criteria (*n* = 48) or declined to participate (*n* = 2) or had difficulty arranging transportation to the study site (*n* = 19). A total of 44 patients were randomly assigned to either the EG (*n* = 23) or the CG (*n* = 21). Two patients in the EG and 3 in the CG subsequently withdrew; of the remaining 39 patients, 21 in the EG and 18 in the CG completed the study.

There were no significant between-group differences in demographics and clinical data (Table 1) or in primary and secondary outcome measures at baseline (T0). Cerebellar functions assessed with EDSS subitem were homogeneous between EG and CG and the median score was 1 corresponding to “Abnormal signs without disability.”

**Table 1:** Demographic and clinical characteristics of treated subjects.

	**Experimental Group**	**Control Group**	**Between-group analysis**
	**(*n* = 23)**	**(*n* = 21)**	
Age (years) [mean (SD)]	51.96 (10.87)	50.67 (10.80)	n.s.
Gender (Male/Female)	10/13	41487	n.s.
Disease onset age (years) [mean(SD)]	37.57 (11.91)	36.57 (8.82)	n.s.
Disease duration since diagnosis (years) [mean(SD)]	13.48 (7.82)	14.19 (9.78)	n.s.
Type of MS (RR/PP/SP, respectively)	16/01/06	10/02/09	n.s.
EDSS score [median (Q1–Q3)]	6.00 [5.00–6.60]	6.00 [4.00–7.25]	n.s.
*Cerebellar Functions*	1 [0–1]	1 [0–3]	n.s.
Dominant hand (R/L)	19/4	20/1	n.s.
Trained hand (DH/NDH)	11/12	9/11	n.s.
MAS [median (Q1–Q3)]
*Elbow*	0 [0–1]	0 [0–1]	n.s.
*Wrist*	0 [0–0]	0 [0–0.5]	n.s.
*Fingers*	0 [0–1]	0 [0–1]	n.s.
TSS Score [median (Q1–Q3)]
*Rest tremor*	0 [0–0]	0 [0–0]	n.s.
*Postural tremor*	0 [0–1]	0 [0–1]	n.s.
*Kinetic tremor*	0 [0–1]	0 [0–1]	n.s.

*RR, Relapsing Remitting; PP, Primarly Progressive; SP, Secondary Progressive; EDSS, Expanded Disability Status Scale; DH, Dominant Hand; NDH, Non Dominant Hand; MAS, Modified Ashworth Scale; TSS, Tremor Severity Scale; MI, Motricity Index*.

### Primary outcome

There were no significant between-group differences in ARAT scores (Table 2). Both groups showed an overall significant improvement in performance at T1 and T2 (*p* < 0.001).

**Table 2:** Clinical outcome measures and inferential statistics.

					**Intervention Phase**			**Repeated measures ANOVA**		
		**Before**	**After**	**FU**	**Mean between-group differences**			**Group between-participants**	**Time within-participants**	**Time x group interaction**
					**Before**	**After**	**FU**			
**Outcome Measure**	**Group**	**Mean (SD)**	**Mean (SD)**	**Mean (SD)**	**95% CI (LB, UB)**	**95% CI (LB, UB)**	**95% CI (LB, UB)**	***p***	***p***	***p***
***PRIMARY OUTCOME MEASURE***										
ARAT	EG	41.57 (15.22)	46.52 (14.43)	45.76 (15.55)	0.02 (−9.69; 9.72)	1.41 (−7.63; 10.45)	0.32 (−9.31; 9.94)	n.s.	<0.001^*^	n.s.
	CG	41.55 (14.62)	45.11 (13.39)	45.44 (14.01)						
***SECONDARY OUTCOME MEASURES***										
NHPT speed	EG	0.19 (0.10)	0.21 (0.11)	0.22 (0.12)	−0.004 (−0.07; 0.06)	−0.02 (−0.10; 0.06)	−0.02 (−0.10; 0.06)	n.s.	<0.001^*^	n.s.
	CG	0.19 (0.10)	0.22 (0.13)	0.24 (0.13)						
FM	EG	51.71 (14.89)	54.56 (13.71)	55.61 (13.41)	−0.95 (−9.88; 7.97)	−2.55 (−10.65; 5.56)	−0.50 (−8.87; 7.87)	n.s.	<0.001^*^	n.s.
	CG	52.67 (12.63)	57.11 (11.27)	56.11 (12.38)						
MI	EG	81.24 (17.11)	84.94 (14.20)	85.32 (14.69)	0.52 (−9.59; 10.63)	0.22 (−8.72; 9.15)	1.15 (−8.21; 10.51)	n.s.	0.001^*^	n.s.
	CG	80.72 (14.04)	84.72 (13.31)	84.17 (14.10)						

EG, Experimental group; CG, Control group; FU, Follow Up; ARAT, Action Research Arm Test; NHPT, Nine Hole Peg Test; MI, Motricity Index.

* Statistically significant.

° *Trend toward statistical significance; for post-hoc analysis p significant if <0.025; 95% confidence interval (CI), lower bound (LB), upper bound (UB)*.

### Secondary outcomes

**Figure 4:**
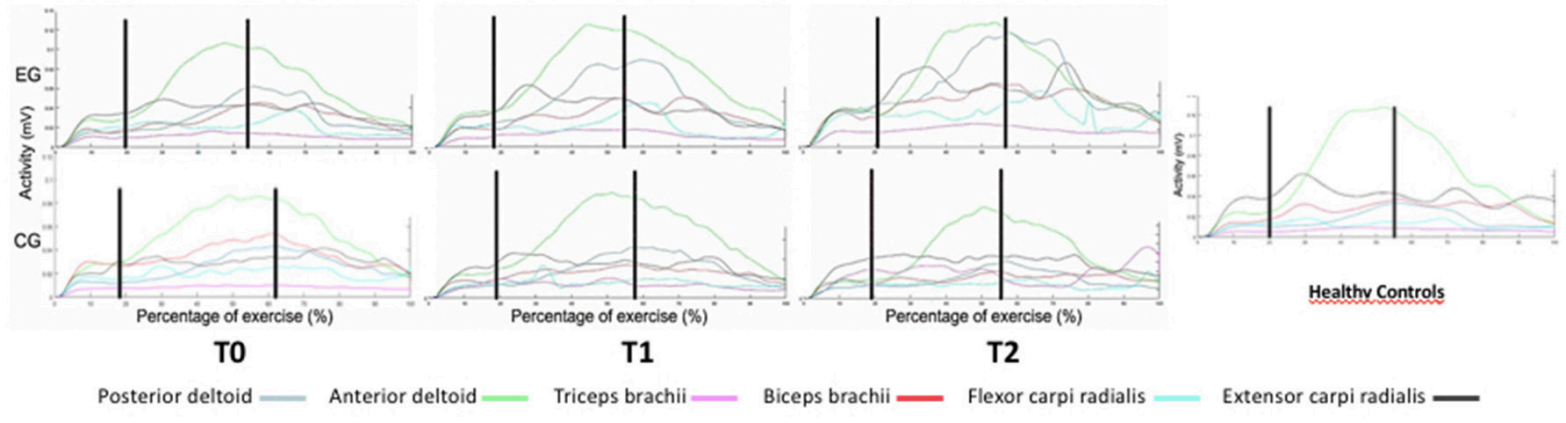
EMG muscle activity during the reach-to-grasp task.

No adverse events or safety concerns arose during the conduction of study. Both groups showed an overall significant improvement on all secondary outcome measures without significant between-group differences (Tables 2, 3). Both groups presented at the enrollment a notable capacity at the UL FMA (29). Only the EG showed significant changes in the motor activity log-amount of use (MAL-AOU) at both T1 and T2 (Table 3). At T2 significant improvements in muscle strength during finger extension (*p* = 0.02) and flexion (*p* < 0.001) were noted in the EG. Preliminary observation of muscular function revealed abnormalities in lifting the wooden cube for both groups. After training, the EG showed increased extensor carpi activity similar to activation in healthy control subjects. These affects were maintained at the follow-up evaluation (Figure 4).

**Table 3:** Clinical QOL, participation outcome measures and inferential statistics.

					**Intervention Phase**			**Repeated measures ANOVA**			***Post hoc*** **analysis**	
		**Before**	**After**	**FU**	**Mean between-group differences**			**Group between-participants**	**Time within-participants**	**Time x group interaction**	**within-group**	
					**Before**	**After**	**FU**				**T1-T0**	**T2-T0**
**Outcome Measure**	**Group**	**Mean (SD)**	**Mean (SD)**	**Mean (SD)**	**95% CI (LB, UB)**	**95% CI (LB, UB)**	**95% CI (LB, UB)**	***p***	***p***	***p***	***p***	***p***
MAL-AoU	EG	96.31 (45.10)	100.71 (46.15)	100.92 (46.71)	−6.00 (−34.83; 22.84)	−4.07 (−32.87; 24.73)	−3.69 (−32.97; 25.60)	n.s.	0.007^*^	n.s.	0.005^*^	0.016^*^
	CG	102.31 (43.58)	104.78 (42.55)	104.61 (43.45)							n.s.	n.s.
MAL-QoM	EG	96.95 (41.37)	102.15 (42.17)	101.68 (43.30)	1.01 (−25.44; 27.45)	1.43 (−25.76; 28.62)	1.79 (−25.99; 29.57)	n.s.	<0.001^*^	n.s.	<0.001^*^	0.015^*^
	CG	95.94 (39.95)	100.72 (41.38)	99.89 (42.10)							0.013^*^	n.s.
MSQOL-54 (PH)	EG	42.46 (19.18)	44.77 (18.60)	45.59 (19.84)	−1.39 (−13.70; 10.92)	1.59 (−11.10; 14.27)	3.21 (−9.18; 15.60)	n.s.	n.s.	n.s.	–	–
	CG	43.85 (17.17)	43.18 (18.75)	42.48 (17.78)							–	–
MSQOL-54 (MH)	EG	54.61 (25.77)	58.78 (22.10)	58.85 (24.29)	−2.14 (−17.50; 13.23)	−1.99 (−17.40; 1.41)	2.29 (−13.52; 18.11)	n.s.	n.s.	n.s.	–	–
	CG	56.75 (19.38)	60.78 (23.19)	57.41 (23.30)							–	–
LIFE-H (acc.)	EG	7.22 (1.90)	7.42 (1.84)	7.36 (1.83)	−0.24 (−1.28; 0.81)	−0.13 (−1.13; 0.87)	−0.23 (1.23; 0.76)	n.s.	0.01^*^	n.s.	0.002^*^	0.026°
	CG	7.45 (1.29)	7.55 (1.22)	7.6 (1.21)							n.s.	n.s.
LIFE-H (PS)	EG	3.96 (0.83)	4.29 (0.65)	4.29 (0.75)	−0.17 (−0.70; 0.36)	−0.09 (−0.54; 0.35)	−0.12 (−0.60; 0.37)	n.s.	<0.001^*^	n.s.	0.001^*^	0.001^*^
	CG	4.13 (0.78)	4.38 (0.71)	4.41 (0.74)							0.001^*^	0.024^*^

EG, Experimental group; CG, Control group; FU, Follow Up; QOL, Quality Of Life; MAL-AoU, Motor Activity Log—Amount of Use; MAL-QoM, Motor Activity Log—Quality of Movement; MSQOL-54 (PH), Multiple Sclerosis Quality of Life (Physical Health); MSQOL-54 (MH), Multiple Sclerosis Quality of Life (Mental Health); LIFE-H (acc.), Life Habits (accomplishments); LIFE-H (PS), Life Habits (Patient Satisfaction).

* Statistically significant.

*°trend toward statistical significance; for post-hoc analysis p significant if <0.025; 95% confidence interval (CI), LB lower bound, UB upper bound*.

## Discussion

The main finding of this RCT is that upper limb activity and function improved after both robot-assisted hand training and robot-unassisted treatment in these patients with MS. Interestingly, only the group that received robot-assisted hand training reported significant improvements in the use of the treated upper limb and in the assessment of skills in the life habits domain (accomplishments). In addition, preliminary observation of muscular activity showed enhancement of extensor carpi activation only in the robot-assisted hand training group, suggesting a task-specific effect of this training mode on muscle activity.

Upper limb robot-assisted interventions have been increasingly applied in neurorehabilitation because they offer the advantage of high-intensity training, volume, and duration that can be delivered without the constant presence of a physiotherapist. However, several questions still need to be addressed: optimal therapy content and dosage according to degree of upper limb disability, effectiveness of rehabilitation strategies in improving upper limb function, and influence of type of training approach on increasing upper limb capacity and performance (8).

In a pilot RCT study conducted by Carpinella et al. 22 patients with MS were randomly assigned to receive either a robot-assisted reaching task (RT) training or robot-based training (RMT) in which objects had to be grasped and manipulated (Braccio di Ferro, Celin srl, La Spezia, Italy) (9). After 8 sessions, a significantly larger improvement in grasping was observed in the group assigned the RMT protocol. In a pilot study conducted by Feys et al. the effects of additional robot-supported virtual learning training added to conventional treatment were investigated (10). Seventeen patients received either 3 weekly sessions of conventional training alone or conventional training plus training with a 3-DoF haptic device (Haptic Master training, MOOG, the Netherlands). After 8 weeks, no significant changes in function and activity level were reported in either the intervention or the control group. However, a near-threshold significant between-group difference was measured for the MAL (amount of use and total). In their feasibility cross-over study, Vergaro et al. tested a robotic therapy protocol for rehabilitation of poor coordination in 8 patients with MS (30). No significant differences in the Nine Hole Peg test score were observed after administration of 8 training sessions of the two training protocols, though the movements became smoother after training.

While differences in sample and methods hamper comparisons between our and previous studies, our results provide evidence for a restorative potential of upper limb function after specific rehabilitation interventions in patients with MS. Both training protocols were administered to improve impairments in upper limb function (and hand dexterity), which would explain why no significant differences were detected between the two interventions. Training specificity differed slightly between the two approaches: the robot-assisted hand training was mainly focused on visual feedback and a task-specific approach, whereas the robot-unassisted training dealt with functional movement and context-specific training (3). Both training protocols shared common features such as unilateral training, mobility, stretching, and exercise progression (3). However, only with the more intensive, repetitive, and task-specific training did the amount of upper arm use and muscle activity improve, and continue through to follow-up assessment.

Chronic upper limb disuse might contribute to disability and explain the functional differences beyond the adaptive functional reorganization observed in clinical stages and forms of MS (2). Patients with MS may develop a negative learning phenomenon, which consists of relying on their less affected arm to perform activities of daily living with a progressive suppression of movements in the more affected arm (2, 25). Interference with this learned phenomenon may be the mechanism through which physical therapy can limit the extent of upper limb disability in stroke patients and in patients with MS (31–33).

**Figure 5:**
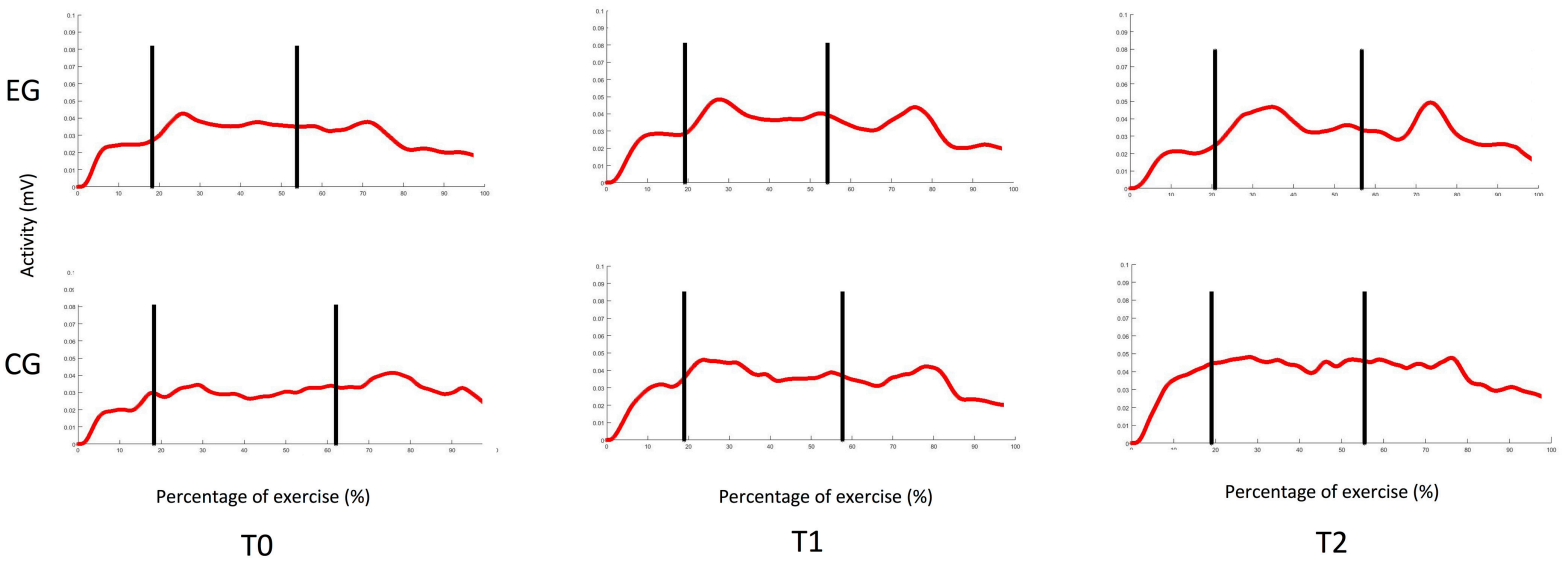
EMG muscle activity of the Extensor Carpi Radialis.

Unfortunately, we were unable to identify the factors related to robot-assisted training that would have been crucial to this finding. Nevertheless, we argue that such training may boost more successful use of the hand that, in turn, could increase confidence in performing upper limb activity. The role of training intensity, along with the need to better balance uni- and bimanual upper limb exercise, may have contributed to successful outcomes. The visual feedback provided by the Amadeo system may have increased patient motivation during training and awareness of upper limb capacity Moreover, training intensity may have reduced the one's perception of difficulty and assistance required by the patient during upper limb activity assessed with LIFE-H-accomplishment section. Although both groups presented a notable capacity of the UL and minimal signs of cerebellar dysfunctions, the interactive feedback provided by the robotic training was a stimulating way to increase patient motivation, reduce effort and increase performance during ADLs (29). This finding suggests that the robotic training effects may be extended beyond the physical aspects. Literature supports a disagreement between MS patients and treating physician regarding factors affecting the quality of life (34). The changes observed in EMG activity may support this view and are in line with previous preliminary reports (35). A smaller difference between the maximum and the minimum of extensor carpi radialis (ECR) activation as compared with healthy subjects was observed after treatment and only the EG recovered ECR activity, improving its modularity. One possible explanation for this is the specific training of finger muscles, and wrist stabilizer muscles indirectly, with the Amadeo system (Figure 5).

The strengths of the present study are the relatively large patient sample and the low drop-out rate, which suggest the feasibility of robotic training in patients with MS. The comprehensive and multidisciplinary evaluation of upper limb disorders according to the ICF framework, and the EMG analysis using a standardized experimental protocol to investigate the training effects on muscle activity are further strengths of this study. The study limitations are the lack of patient stratification by degree of impairment, the lack of assessment of cognitive and mood decline and the lack of neuroimaging support.

To conclude, as a part of the multifaceted management of upper limb rehabilitation, robotics is a feasible and valid approach to improving upper limb function and enhance UL use in patients with MS. Robotics holds promise and potential to enrich rehabilitation care in MS, but issues such as optimal dosage according to degree of upper limb disability need still to be addressed.

## Ethics statement

All procedures performed in the study were in accordance with ethical standards of the IRB and with the 1964 Helsinki declaration and its later amendments or comparable ethical standards. The Institutional review board approval for the study was provided by the AOUI of Verona (prog n.230CESC).

## Author contributions

MG and NS have made substantial contributions to conception and design. AG, MB, MatC, MarC, EC, and AP participated in the enrollment phase. JC and CD carried out the clinical assessment. NV, MF, and ED carried out the Instrumental assessments. SM and EB designed the algorithm for EMG data analysis. MG and NV participated in the statistical analysis and drafted the manuscripts. NV, FF, AW and LS participated in the manuscript revision process and gave the final approval of the version.

### Conflict of interest statement

The authors declare that the research was conducted in the absence of any commercial or financial relationships that could be construed as a potential conflict of interest.
